# Multiple-beam and double-mode staggered double vane travelling wave tube with ultra-wide band

**DOI:** 10.1038/s41598-020-77204-w

**Published:** 2020-11-19

**Authors:** Zheng Zhang, Cunjun Ruan, Ayesha Kosar Fahad, Chenyu Zhang, Yiyang Su, Pengpeng Wang, Wenlong He

**Affiliations:** 1 School of Electronic and Information Engineering, Beihang University, Beijing, 100191 China; 2 Beijing Key Laboratory for Microwave Sensing and Security Applications, Beihang University, Beijing, 100191 China; 3 College of Electronics and Information Engineering, Shenzhen University, Shenzhen, 518061 China

**Keywords:** Electrical and electronic engineering, Electronics, photonics and device physics

## Abstract

This paper presents design, fabrication and cold test of an ultra-wide band travelling wave tube (TWT) with planar alignment multiple pencil beams. The fundamental double-mode of staggered double vane slow wave structure (SDV-SWS) rather than the only one mode are put forward and adopted to match with the same electron beam to increase the bandwidth greatly. Simultaneous planar alignment multiple pencil beam tunnels are designed to improve interaction impedance and then to enhance output power, gain, efficiency, growth rate. The transmission performance of a two-stage 51-period SDV-TWT in G-band with structure attenuator between two sections shows that it indeed has an ultra-wideband performance from 81 to 110 GHz. By using computer numerical control machining, the SDV-SWS was manufactured and a detailed cold test was conducted. Good agreement is found at the wide band, where S_21_ is above − 5 dB and S_11_ is below − 10 dB. 3D PIC simulations with double-mode multiple-beam SDV-TWT within total length of 70 mm show that it can get a nearly 2120 W peak output power, a 42.5 dB corresponding gain and a 10.7% electron efficiency at 94 GHz with a 22.1 kV beam voltage and a 3 × 0.15A beam current. The 3 dB bandwidth of our double-mode SDV-TWT can achieve about 29 GHz.

## Introduction

Recently, millimeter and terahertz technology has received great interest due to their potential applications in diverse modern scientific fields including advanced telecommunication systems, high-resolution radars, biomedical imaging and sensing^1–3^. Much of attention has been paid to vacuum electronic devices (VEDs) and vacuum integrated amplifier technologies, especially for their capability of producing high power and high efficiency with thermal robustness and reliability in a compact package^4,5^. Travelling wave tubes (TWTs) have been demonstrated to be one of the most significant amplification devices of all the VEDs which can provide high output power in a broadband^6,7^. Slow-wave structures (SWSs), as a key part of TWTs influence the performance considerably, so the investigation of SWSs has always been an important section to meet the design requirements of TWTs in millimeter and terahertz band. Up to now, many new types of planar SWSs such as folded waveguide (FWG)^8^, sine waveguide^9^ and staggered double vane (SDV)^10^ have been presented to solve the issues caused by increased working frequency. The size of SWSs becomes smaller which makes the traditional fabrication technique hard to meet matching tolerance requirement, and the traditional helix-TWTs and coupled-cavity TWTs cannot get a high output power and a wide band at millimeter and terahertz band. Thus, these new types of planar SWSs really have advantages that they can be easily fabricated by using 2D plane manufacturing technology with the development of microelectron mechanical systems, e.g. computer numerical control (CNC) and UV-LIGA.

Comparison with the widely used planar FWG, the SDV-SWS have been proved to achieve high power, high efficiency, high growth rate, high interaction impedance, and wideband compatibly with sheet beam at W-band and G-band^11–14^. A design study using SDV-SWSs combined with a sheet beam has shown that an output power of over 1 kW should be possible from 90 to 95 GHz^15^. However, the formation and transportation of such a micro-miniature intense sheet electron beam can be really difficult particularly in beam channel with sub-millimeter structures, and long transmission distance with high current density. To avoid these problems, planar alignment multiple pencil beam SDV-SWSs have been designed with a 2250 W output power and a 15 GHz bandwidth^11^. Not only can pencil beam TWTs give the good solution to above issue, but they have additional advantages of simple RF input/output coupler design compared to sheet beam SDV-TWT, for the RF signal which is naturally cut-off in pencil beam tunnel. Recently, the significant progress for the fabrication and measurement of sheet bam SDV-TWT is performed in UC-Davis in 2017^4^, the experimental results show that the 3 dB bandwidth of 14 GHz with output pulse power of 11 W, and 3 dB bandwidth of 6 GHz with output pulse power of 107 W have been achieved with the fabricated SDV-TWT in G-band. However, the improvement of bandwidth for SDV-TWT amplifier is still under potentials with quite a long and difficult way to go in this field.

In this paper, to further improve the bandwidth, keep the high output power and avoid the serious problems of sheet beam formation and long distance transportation, instead of using the only fundamental single mode to match with the electron beam, the fundamental double-mode SDV-SWS with planar alignment three-pencil beam in W-band is studied thoroughly. It is found that the beam-wave matching band indeed increases as twice compared to our previous work^11^. Also, the interaction impedance is also about two times higher than that of sheet beam SWS in the same band. Then, a simple coupler is used to get a good transmission performance, and a two-stage SWS structure is also designed to suppress the reflection and oscillation for the SDV-TWT. Moreover, the two stage SDV-SWS for W-band TWT is fabricated by the CNC machining system, and a cold test is conducted by using a vector network analyzer, good agreements are achieved compared with the simulation results. Finally, 3D PIC simulation shows that it can obtain a high output power of nearly 2120 W at 94 GHz with a two-stage 51-period SWS-TWT, the gain of 42.5 dB and the efficiency 10.7%. The 3 dB bandwidth about 29 GHz from 81 to 110 GHz can be achieved, which is almost twice than that of our previous work (15 GHz)^11^. Thus, our double-mode, two-stage and planar alignment three-pencil beam SDV-TWT indeed has good performances of high power and broadband in millimeter and terahertz band.

## High-frequency characteristics

**Figure 1:**
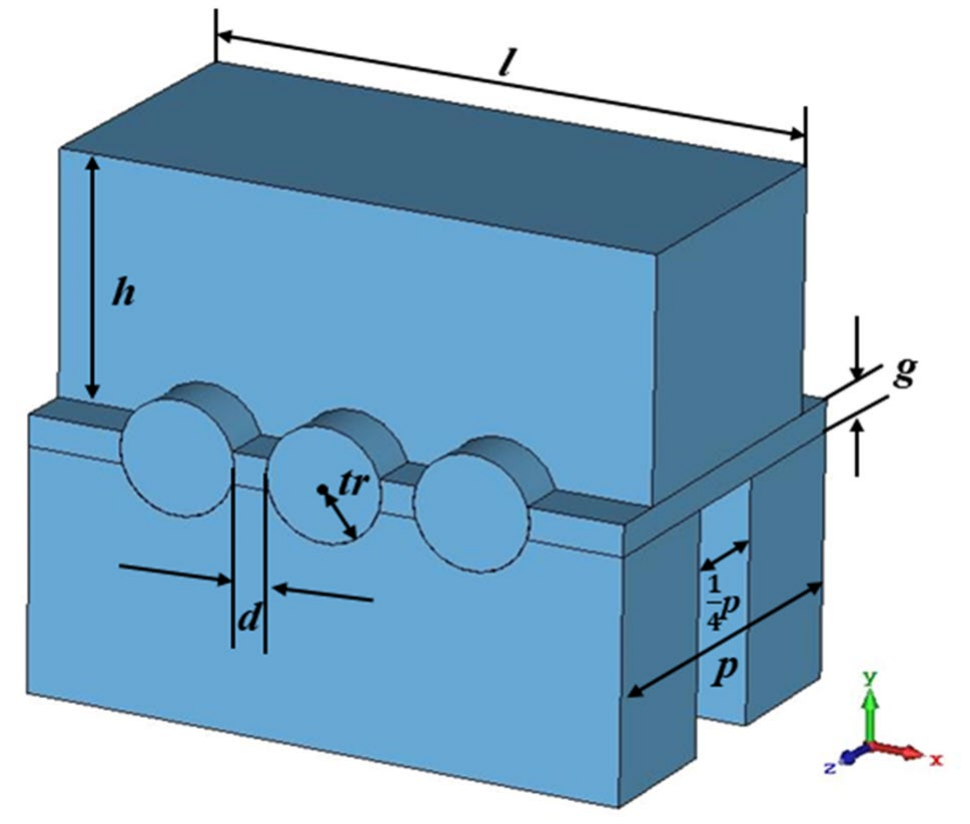
3-D schematic of a single period of SDV-SWS. It is a physical inverse model, where the blue part is a vacuum section and the background is the actual material like copper.

A single period of staggered double vane slow-wave structure is given in Fig. 1, which can form a strong symmetric axial electric field distribution along the direction of electron movement^16^. Also, compared to sheet beam, the planar alignment pencil beam tunnels can constrain the electric field better to increase the axial electric field^11,17^. So, sheet beam tunnels are replaced by three pencil beam tunnels as is shown in the figure, where ***l*** is the width of the vane, ***h*** is the height of the vane, ***p*** is the length of the single period SDV-SWS, ***g*** is the distance from the upper vane to the lower vane, ***tr*** is the radius of the pencil beam tunnel and ***d*** is the distance between the edges of two adjacent beam tunnels.

### Dispersion characteristics

In SWS, the fundamental mode (TE_10_ mode) is designed to interact with electron beam. Due to the symmetry of staggered double vane structure, solutions of the dispersion equation of electromagnetic field can be divided into two cases. One is the odd mode corresponding to mode 1 in Fig. 3, the other is the even mode corresponding to mode 2. So mode 1 and 2 are both belong to the fundamental mode. The central frequency and the synchronous voltage for the SDV-TWT are set to ***f***_***0***_ = 94 GHz and ***V***_***0***_ = 22 kV respectively. We choose phase shift ${\varvec{\phi}}$=2.7π to avoid the lower cutoff oscillation^18^ and to match the 22 kV electron beam with Brillouin curves of mode 1 and mode 2 as much as possible, which means we hope our SDV-SWS operates at the first double-mode which are designed to promote the bandwidth. The detail analysis will be given in Fig. 3. Thus, we can calculate the value of synchronous phase velocity and the length of the single period SDV-SWS ***p*** by using (1) and (2), where ***v***_***p***_ is the phase velocity of electrons, ***c*** is the speed of light in vacuum.

1 $${v}_{p}=\beta c=c\sqrt{1-\frac{1}{{(1+{V}_{0}/5.11\times {10}^{5})}^{2}}}=0.284c$$

2 $$p=\frac{\phi }{{k}_{z}}={v}_{p}\frac{\phi }{\omega }={v}_{p}\frac{\phi }{2\pi {f}_{0}}=1.22\mathrm{mm}$$

**Figure 2:**
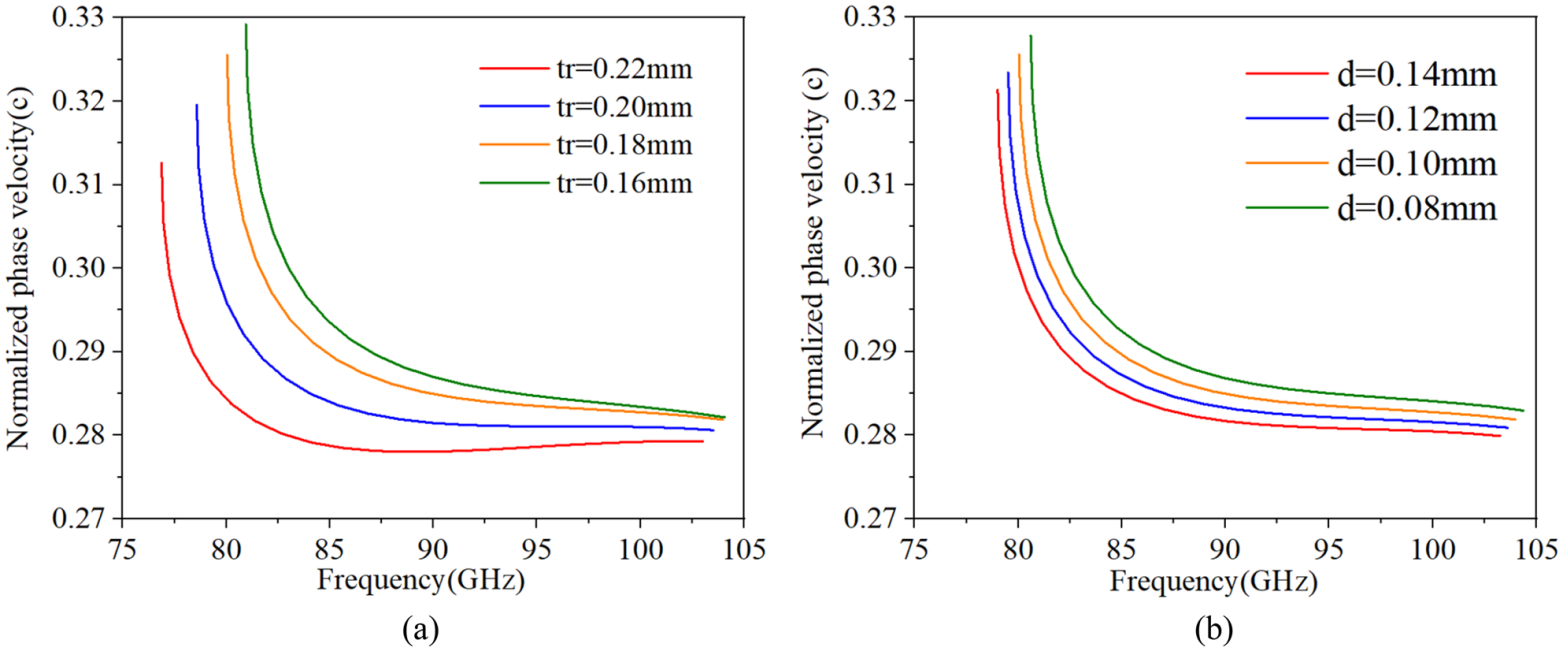
Effects of* tr* (**a**) and ***d*** (**b**) on dispersion characteristics.

Then, the effects of each structure parameters on dispersion characteristics for SDV-SWS can be studied thoroughly, and the results are similar to our previous work^11^. As ***l*** increases, the whole phase velocity decreases; as ***h*** increases, the phase velocity of high frequency decreases but that of low frequency keeps the same. Then, when ***tr*** increases, we can regard it as the width of the vane ***l*** increases and the height of the vane ***h*** decreases, so the whole phase velocity decreases but the decrease in phase velocity of low frequency is more than that of the high frequency as is shown in Fig. 2a. Next, as ***d*** increases, like the width of the vane ***l*** increases, the whole phase velocity decreases as is shown in Fig. 2b. Structure parameter ***g*** has little effect on dispersion characteristics but has a great effect on interaction impedance and transmission characteristics.

**Figure 3:**
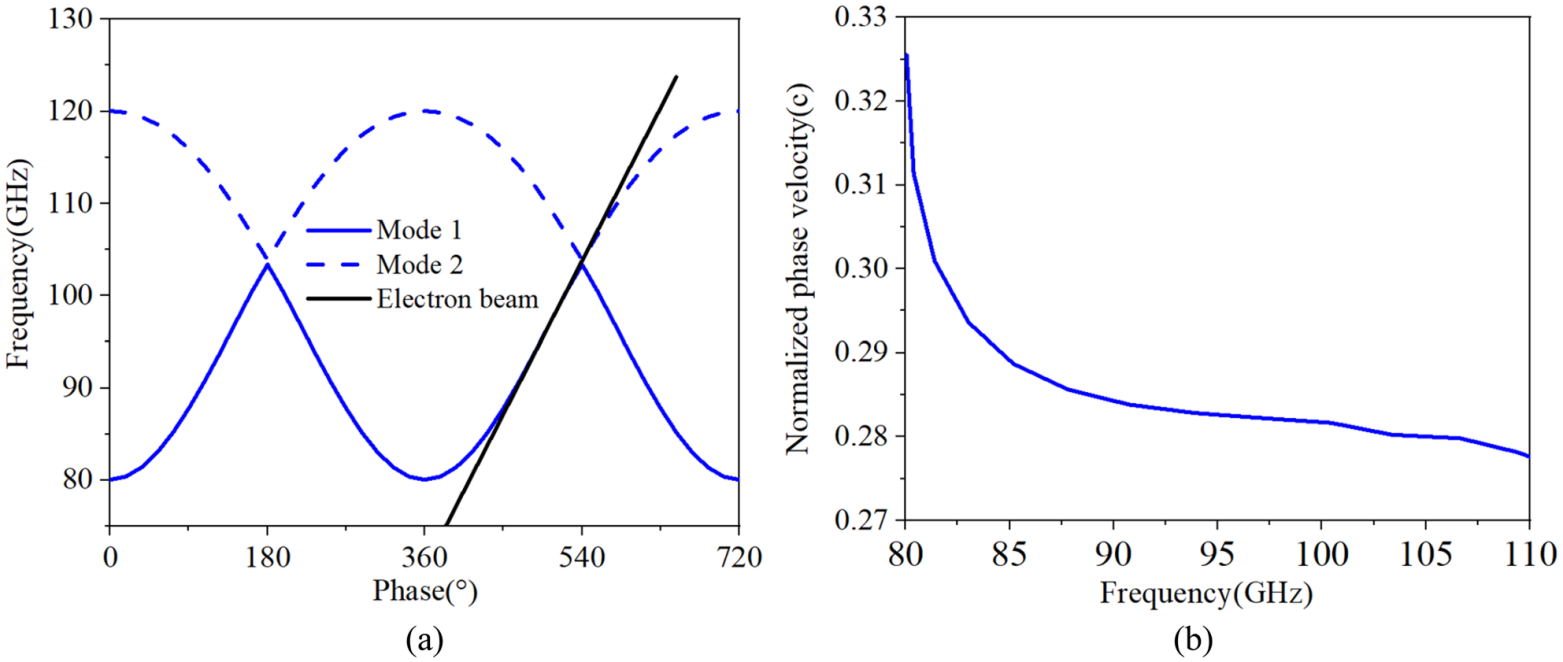
The dispersion characteristics of SDV-SWS, Brillouin curves (**a**) and normalized phase velocity (**b**), where mode 1 from 80 to 103 GHz and mode 2 from 104-120 GHz match the same electron beam line.

Considering the influence of dispersion, interaction impedance, beam current and fabrication, we determined and optimized the structure parameters which are given in Table 1. Then, the final optimized dispersion characteristics is shown in Fig. 3. We can see that the SDV-SWS can be operated in the fundamental double modes, beam-wave velocity matching band is good enough to reach 29 GHz from 81 to 103 GHz (mode 1) , and 104 GHz to 110 GHz (mode 2). It is indicated that, we can use the same electron beam to interaction with these two modes, which may promote the bandwidth for the beam-wave interaction in the design of SDV-TWT. There is a small band gap between mode 1 and mode 2, the gap is from 103.4 to 103.9 GHz and it is possible to generate self-excited oscillation in this narrow band gap due to high impedance. However, the TWT device we designed has a ultra-wide band from 81 to 110 GHz. The gap between two modes are very small comparing to the ultra-wide band. Certainly, it is best to avoid using 103–104 GHz frequency band in the real engineering work in case of oscillations. Besides, we have conducted the hot test simulation to calculate the output signal of each frequency carefully, especially at the frequency of 103 GHz and 104 GHz. The background material is set to copper, and the results show that there is no any oscillation at this two frequency, the output signal is stable and the frequency spectrum is pure.

**Table 1:** Optimized structure parameters.

Parameter	Value (mm)
*p*	1.22
*l*	1.86
*g*	0.10
*h*	0.77
*tr*	0.18
*d*	0.10

### Interaction impedance

As the main characteristic for the design of TWT, the interaction impedance ${{\varvec{k}}}_{{\varvec{c}}{\varvec{n}}}$ can determine the gain, efficiency, and growth rate of the tube, which is given as follow.

3 $${k}_{cn}=\frac{{\left|{E}_{zn}\right|}^{2}}{2{\beta }_{n}^{2}{P}_{w}}$$

where ***P***_***w***_ is the power along the *z*-axis for the direction of electron movement, ***E***_***zn***_ is the amplitude of the spatial harmonic component which is synchronous with the electron beam, and ***β***_***n***_ is the *n*th spatial harmonic’s phase constant. So, we can see that ***E***_***zn***_ is a key parameter that determines the interaction impedance directly. Based on our previous work, we find that the value of ***tr*** influences the ***E***_***z***_ at the center of the middle beam tunnel. Moreover, compared to sheet beam tunnels, the pencil beam tunnels have a much stronger ***E***_***z***_. That can be explained as pencil beam tunnels can constrain the electric field better in a smaller space to form the stronger field, so the interaction impedance obtains a big increase^11^.

**Figure 4:**
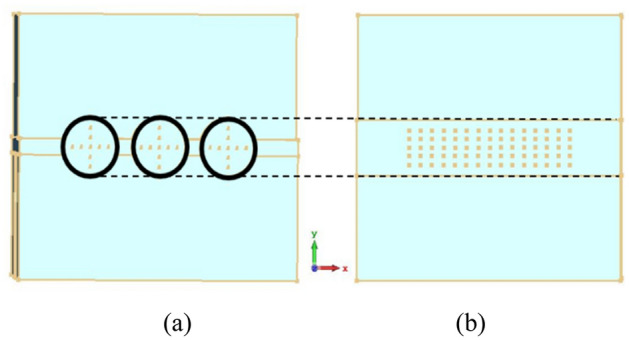
Cross sections of two kinds of beam tunnels, multiple pencil beam tunnels (**a**) and sheet beam tunnels (**b**). The diameter of pencil beam tunnels and the height of sheet beam tunnels are about the same.

**Figure 5:**
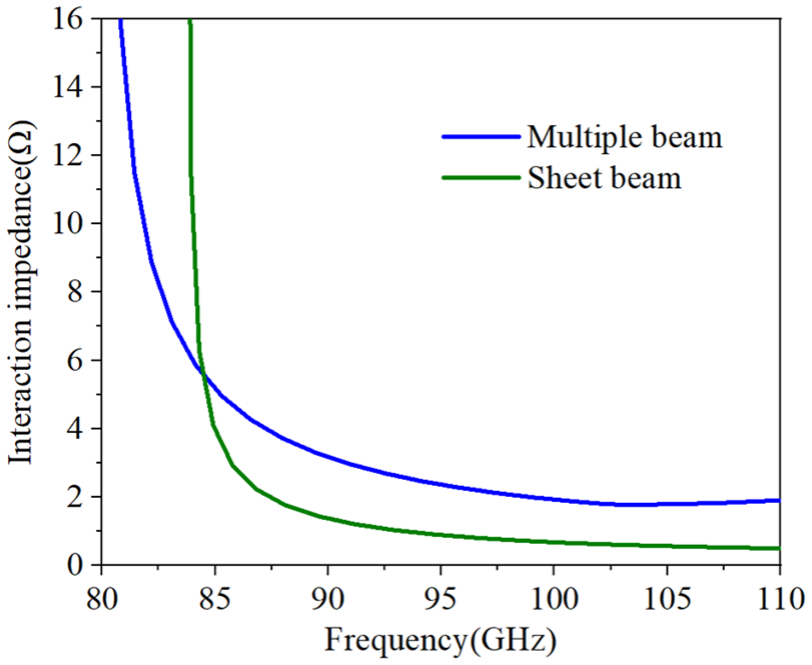
Interaction impedance of two kinds of SWS.

We set the radius of pencil electron beams as ***br*** = 0.12 mm, the average interaction impedance for 9 × 3 points on the multiple pencil beam cross-section and 5 × 15 points on the sheet beam cross-section^19^ can be calculated with the same height of beam tunnel, which is shown in Fig. 4. We can conclude that the interaction impedance of pencil beam SDV-SWS is over 2Ω in beam-wave matching band, which is indeed almost twice higher than that of sheet beam SWS which is over 1Ω as is shown in Fig. 5.

## Transmission characteristics simulation, fabrication and cold test

### Transmission characteristics simulation results

**Figure 6:**
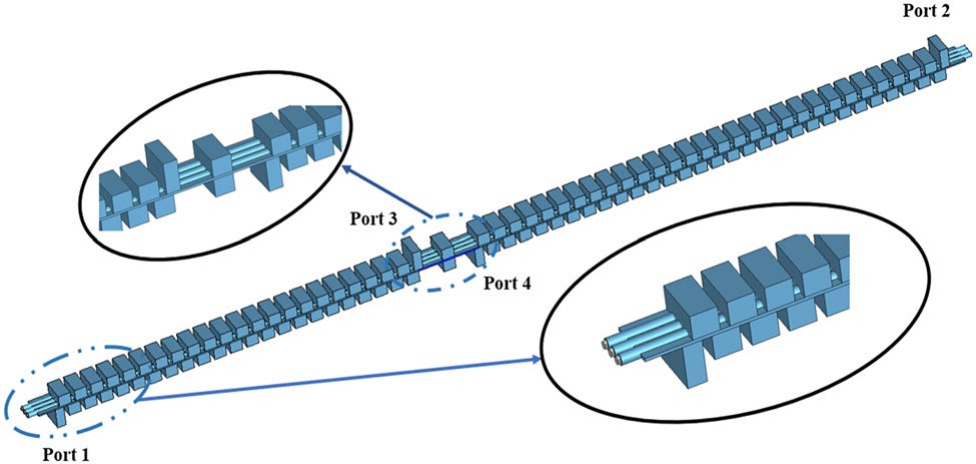
Schematic of two-stage TWT(22 + 29, 51 periods) with attenuator and coupler structure.

**Figure 7:**
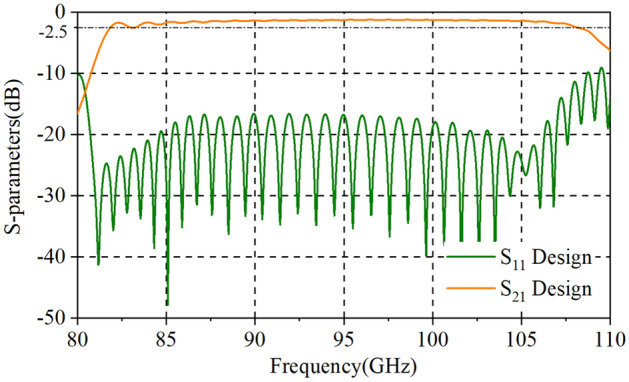
Transmission characteristics of the SDV-SWS together with input and output couplers.

Usually, the RF signal cannot be naturally cut off in the sheet beam tunnel which means an input/output coupler needs to be designed to decrease the reflection of SDV-TWT. However, for our three-pencil beams SDV-TWT, the RF signal is naturally cut off in pencil beam tunnels, so this time we do not need to use a complicated coupler as previous works^20^, instead, a simpler and easier coupler structure was used to be connected to standard rectangle waveguides as is shown in Fig. 6. Besides, a two-stage structure is used as an available way to suppress the oscillation of reflection wave when the number of periods is increased greatly for SDV-SWS, and an attenuator that is used to connect two-stage structures and to suppress the oscillation and reflection is designed. Compared with the single-beam system, the plane multi-beam system could significantly increase the electron beam cross-sectional area and then effectively increase the output power and gain. Although the operating mode is cut off for the electron beam channel, there is still some space between the upper and lower vane which could generate other non-working modes competition like high-order-mode, so we still need an attenuator between Port 3 and 4 to suppress the oscillation and reflection. Considering the fabrication technique of this whole structure and the roughness of metal circuit walls, PEC cannot used as background material for the simulation. Instead, oxygen-free copper was set as background materials and the conductivity was set to 2.25 × 10^7^ S/m in W-band^21^. Using CST’s time domain solver^27,28^, we get the transmission characteristics of our two-stage 51-periods SDV-SWS with first stage of 22 periods and second stage of 29 periods. The simulation results for S-parameters is given in Fig. 7, which shows that the whole structure has a high return loss (S_11_ < − 10 dB) and a low transmission loss in a broad band from 81 to 110 GHz. Just like we designed, this double-mode two-stage multiple pencil beam SDV-TWT has an ultra-wide band of about 29 GHz which is about twice than that of our previous structure^11^.

### Fabrication

To verify our design, we fabricated our SDV-TWT with its SWS by using a CNC precision vertical machining technique which directly mills of circuit structures into bulk Oxygen-free high conductivity (OFHC) copper with two halves. Compared to UV-LIGA, CNC technique increases design flexibility and simplicity. And, different from the structures made of silicon and coated with a thin metal layer, it provides better thermal robustness due to superior thermal and electrical properties. Moreover, compared to additive manufacturing developing recently, it can obtain lower surface roughness^22–25^. Although there are some drawbacks of CNC milling like only one structure can be fabricated at one time and the geometry design is limited^26^, we still chose this CNC milling technique to manufacture our W-band SDV-TWT. Because the number of our circuit structures is with multiple periods and same geometry design, and the rectangular structures can be precisely controlled by CNC.

**Figure 8:**
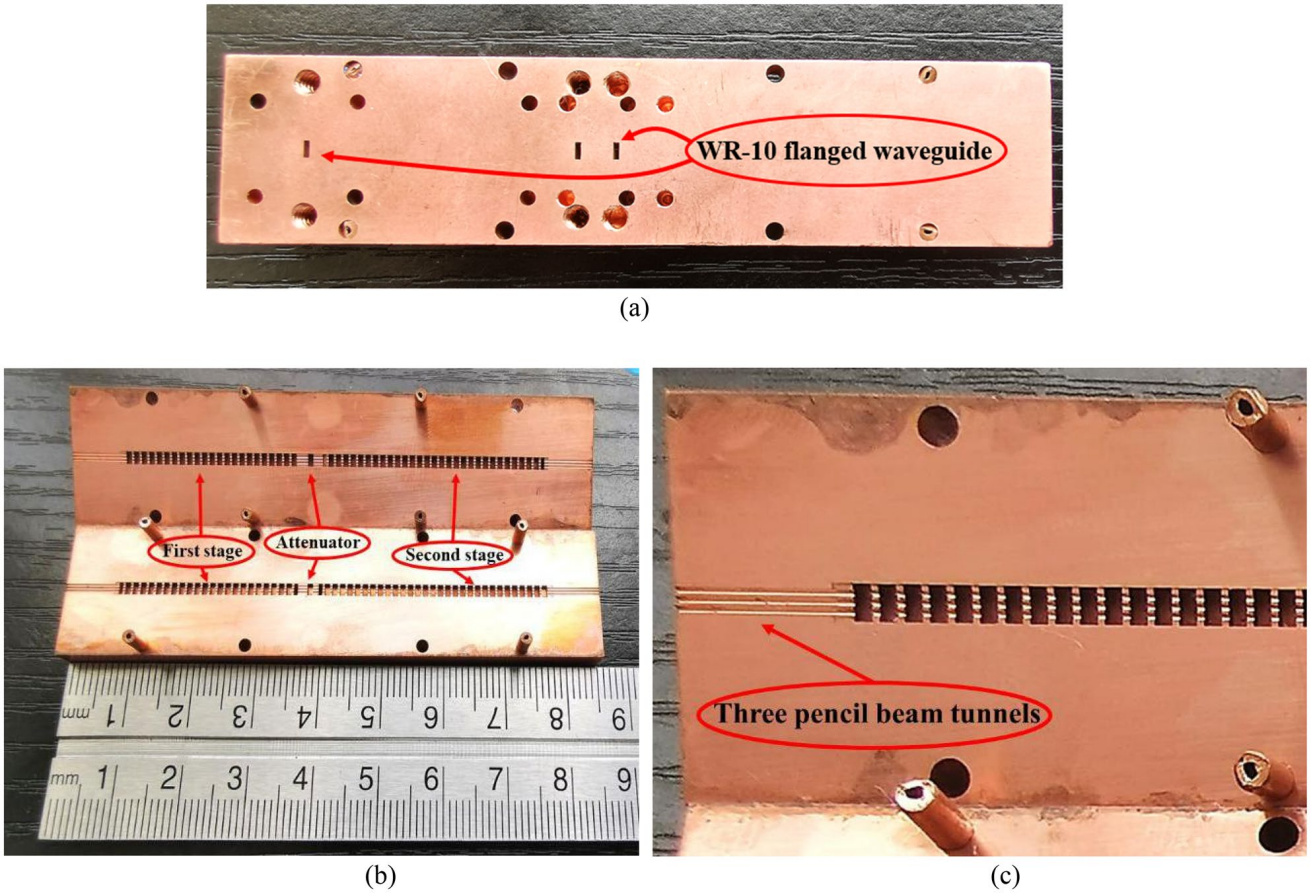
Photos of the fabrication structure outside (**a**), inside (**b**) and (**c**). (**a**) The port in the left is corresponding to Port 1 in **Fig. ****6** and the right one is Port 3. (**b**) Eight pairs of small matching round holes and eight short copper bars are adopted at the edge of the structure. (**c**) Local enlarging photo of three pencil beam tunnels.

Owing to our RF signal input ports which is designed to connect with standard WR-10 flanged waveguide ports directly, the manufacturing structure is simple, and the process of reflection measurements is easy to accomplish. Figure 8a shows images of fabrication structure outside the SDV-TWT where the length, width, and thickness of the whole structure is 85.8 mm, 20 mm and 10 mm respectively. It was machined into two halves through the center of beam tunnels and eight pairs of small matching round holes were designed to further reduce the alignment errors that are drilled at both wide sides. Figure 8b shows images of SDV-SWS and attenuator where the first shorter stage is 22 periods and the second longer stage is 29 periods, which is separated by a 0.9 mm-length and 0.77 mm-height Bragg attenuator. Figure 8c is a partial enlargement of the top left corner of Fig. 8b and shows planar alignment three pencil beam tunnels in detail.

### Cold test and analyses

**Figure 9:**
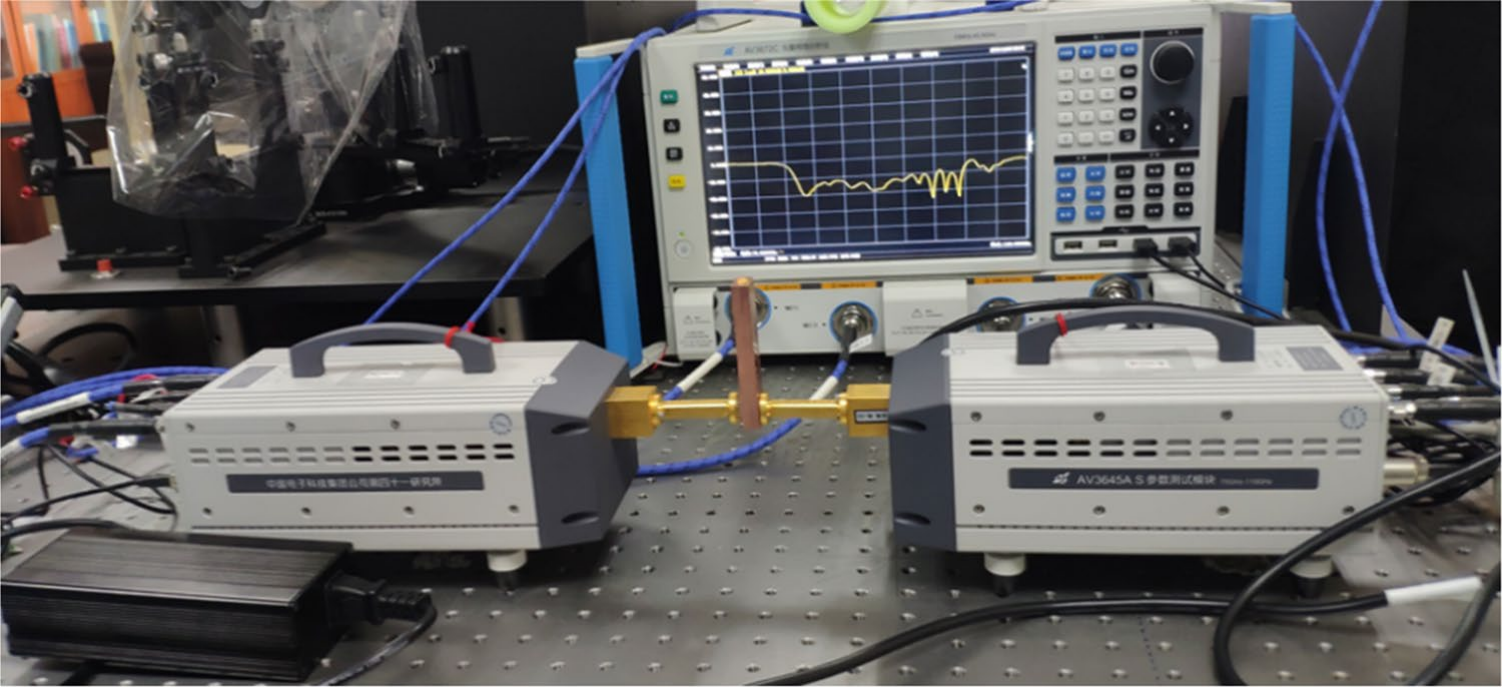
Photos of testing process with a room temperature.

A vector network analyzer (VNA) (AV3672C, 10 MHz–43.5 GHz) which is connected to two frequency extenders (AV3645A) with a frequency range of 75–110 GHz was used for cold test. It utilized a backward wave oscillator (BWO) and can be used for quick testing of MEMS fabricated and CNC machined devices. Test setup for cold test of our SDV-TWT is shown in Fig. 9. Based on our adjusted structure, two-port S-parameter measurements are conducted after careful calibration (AV32141).

**Figure 10:**
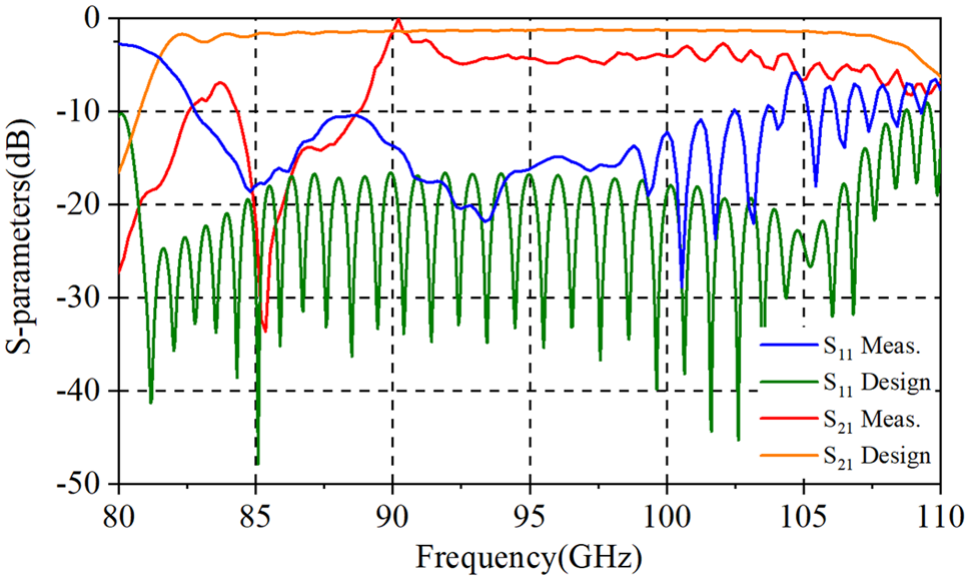
S-parameters results of measurement and design structures.

**Figure 11:**
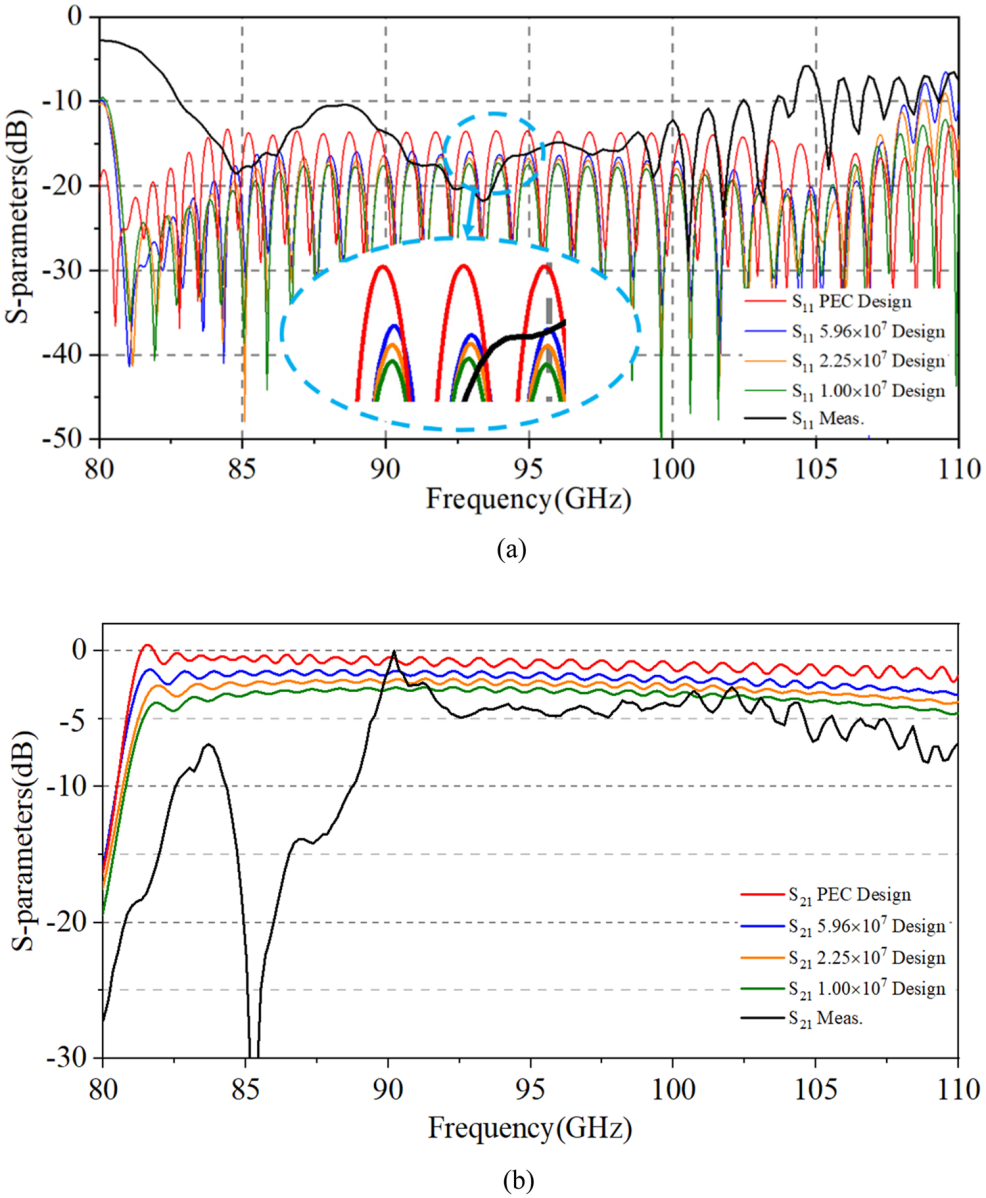
Effects of conductivity on return loss (**a**) and transmission loss (**b**). (**a**) We take the conductivity of PEC as + ∞. The simulation results of S_11_ from top to bottom are + ∞, 5.96 × 10^7^, 2.25 × 10^7^, 1.00 × 10^7^, respectively.

**Figure 12:**
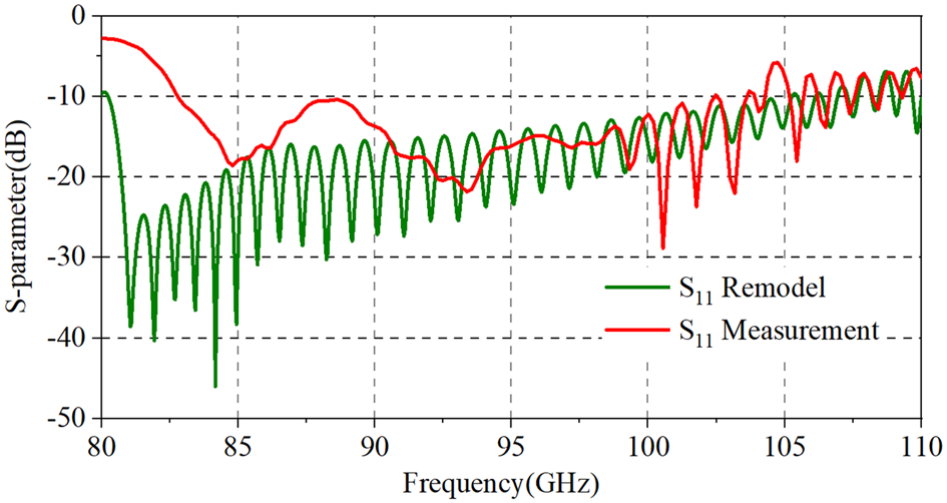
Effects of surface roughness of the SWS ports on return loss.

We conducted several tests to reduce the impact of misalignment issues as much as possible and to get the best results in the process of testing. We tested the return loss (S_11_) and the transmission loss (S_21_) of each port in the two-stage our for fabrication SDV-SWS, and the results are about the same as shown in Fig. 10. It shows S-parameters results, where S_11_ is below -10 dB from 82 to 104 GHz, with a little bit increase from 104 to 110 GHz. And, S_21_ in high frequency band (from 89 to 110 GHz) is above − 5 dB but in low frequency band (from 82 to 89 GHz) is below − 5 dB. Compared to our previous TWT structure, this double-mode two-stage indeed increases the 3 dB bandwidth though it is not as much as simulation results, where the return loss is lower and the transmission loss is higher than that of actual measurement. The main reason may be lies in the deviations of fabrication. We can remodel our TWT simulation structure and calculate the effects to verify our suppositions. The conductivity of actual fabricated parts may be different from that of simulation with the roughness and materials. We calculate several conductivities of materials (+ ∞, 5.95 × 10^7^, 2.25 × 10^7^, 1.00 × 10^7^) as background. The results in Fig. 11 show that as the conductivity decreases, the return loss (S_11_) increases a little but the transmission loss (S_21_) increases a lot in high-frequency band, which means the low conductivity may cause the increases of transmission loss. For the reflection increases, it could be attributed to the surface quality/finish and roughness of the SWS ports which there may be some projections that increases the reflection. To verified this, we have changed the simulation model and calculated the S-parameter results as is shown in Fig. 12. Clearly, the reflection increases in high frequency band. However, for the differences in low frequency band, we account that maybe due to this frequency band from 85-90 GHz is close to the cut-off frequency of the slow wave structure we designed, the transmission loss becomes bad and increases. Besides, there could be a little resonance in the SDV-SWS for much of periods structure at this 85-90 GHz band which causes transmission loss increases remarkably but the return loss almost remains the same. So, we conclude that it is the deviations of fabrication and the roughness of the OFHC copper that caused the differences for the simulation and test results and we really need to pay attention to this in the future processing process..

## Beam-wave interaction simulation results and analyses

To investigate the beam-wave interaction and amplification characteristics of our new double-mode two-stage multiple pencil beam SDV-TWT, we conducted a 3D PIC simulation by using CST^27,28^. At this time, we set background materials to oxygen-free copper whose conductivity is 2.25 × 10^7 ^S/m. The settings of each port and the injection structure of the electron beam are shown in Fig. 6 where the radius of pencil electron beams is ***br*** = 0.12 mm with a filling ration of 44% (the radius of tunnels is ***tr*** = 0.18 mm).

**Figure 13:**
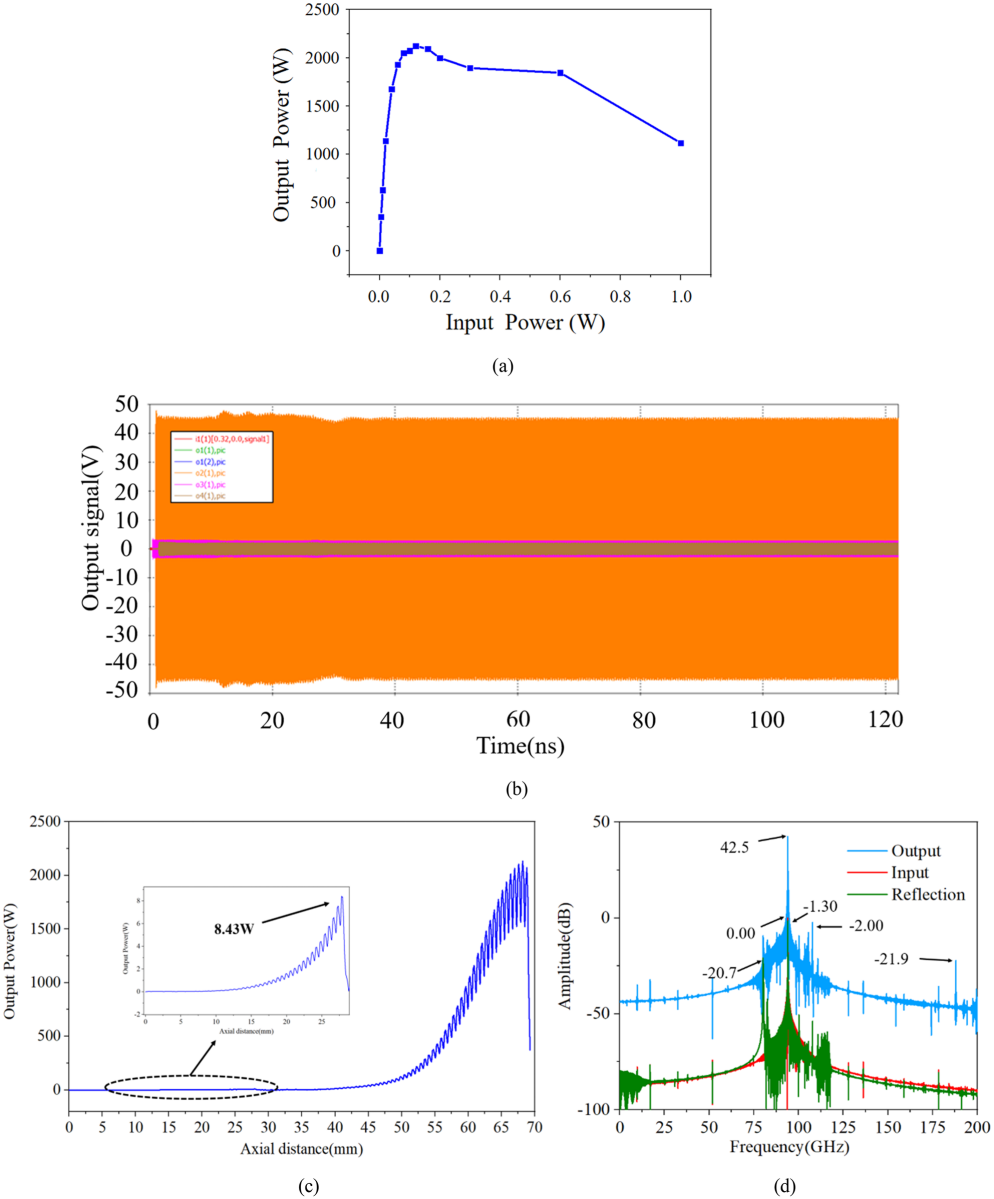
(**a**) Input power versus output power. (**b**) The output signal of the PIC simulation at 94 GHz. The max voltage of output signal is about 46 V, so the output power is over 2120 W with a 0.12 W input power corresponding to a 42.5 dB gain. (**c**) The growth rate of whole SWS. Maximum power reaches 8.43 W before the attenuator then falls, and the growth rate of whole structure is 0.62 dB/mm corresponding to 0.83 dB/period. (**d**) Frequency spectra of the input, reflection and output signal.

Each average current of three pencil electron beams is set as 0.15A with a corresponding current density of 309A/cm^2^, the operation voltage is 22.1 kV and the uniform axial focusing magnetic field is 0.5 T. We set the input power of driving sinusoidal signal at 94 GHz as 0.12 W. We have optimized and adjusted the input power, the output power and gain obtained are saturated as is shown in Fig. 13a. It costs about a week to finish this continuous simulation for one frequency, until 120 ns at a single frequency of 94 GHz with extremely output stability.

As is shown in Figs. 13b,c the 0.12 W input signal is amplified to a 120 ns steady 2120 W output peak signal with a gain of 42.5 dB, an electron efficiency of 10.7% and a growth rate of nearly 0.62 dB/mm, while the power of reflection signal keeps below 0.37 W. It is less than that of the input signal during the whole simulation time. Figure 13d shows the frequency spectrum results, we can see that the maximum monochromatic output signal peaks at 94 GHz which is 42.5 dB higher than that of the input signal while the reflection signal is -1.30 dB lower than that of the input signal which proves that no oscillations are generated. Based on Pierce theory, we know that as the interaction impedance improves the interaction efficiency increases and our multiple pencil beam TWT can get a 10.7% electron efficiency which is much higher than that of sheet beam TWT (5.4%) and single pencil beam TWT (2.8%), so we can get a higher output peak power and a higher growth rate SDV TWT. Besides, compared to the sheet beam SWS (119.4 mm length^19^) and the single pencil beam TWT (102.3 mm length^15^), our multiple pencil beam SWS does not need complicated coupler which means it can not only make the whole structure shorter (70.3 mm) but also reduce the problems of fabrication.

**Figure 14:**
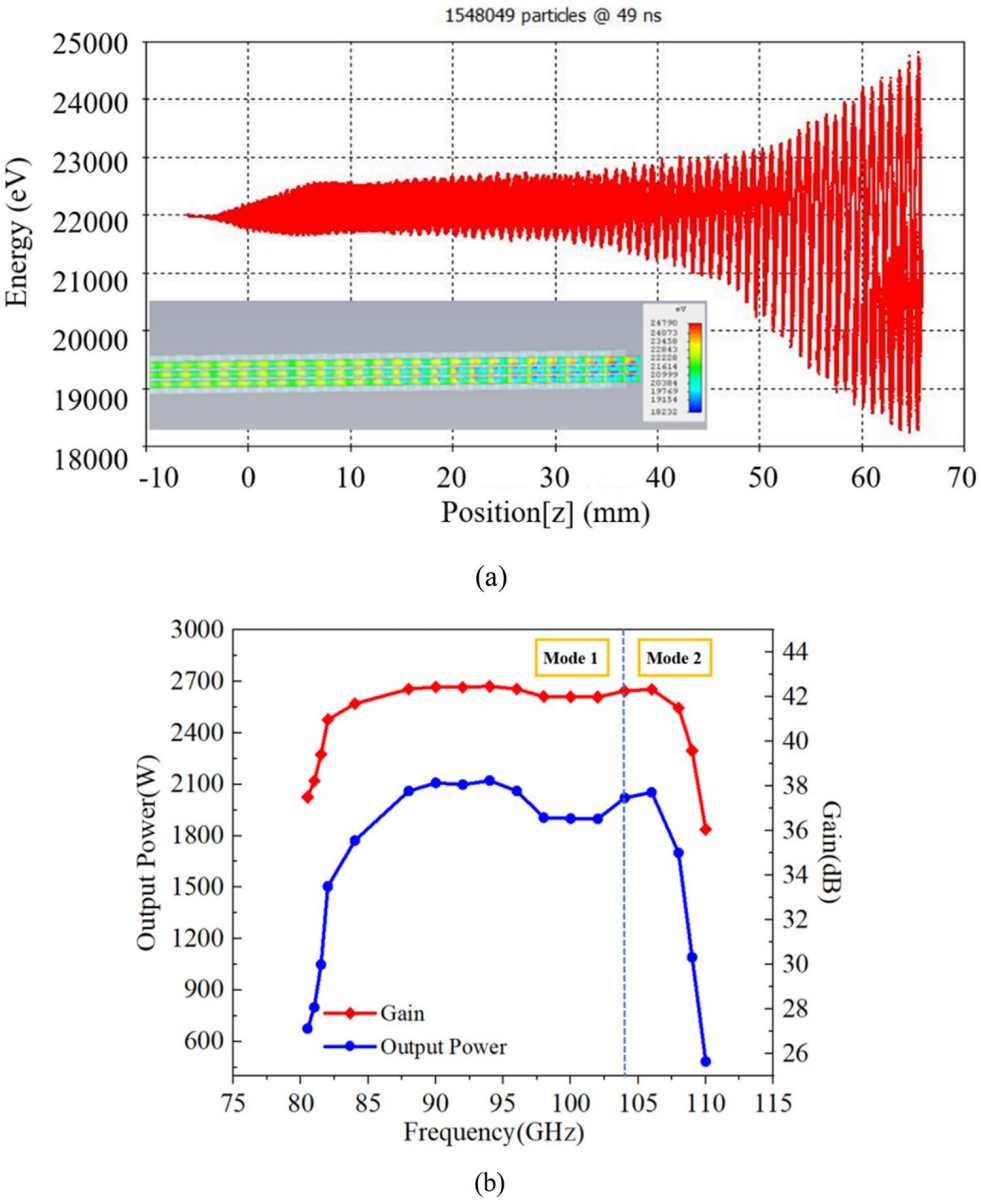
(**a**) Phase space plot of the bunched electron beam at 50 ns. The lower left inset is the electron bunching at the end of the SWS at 50 ns. (**b**) Output power and gain versus frequency.

The phase space plot of the bunched electron beam at 50 ns is shown in Fig. 14a. With initial electron energy of 22 keV, the highest increases to ~ 24.8 keV while the lowest decreases to ~ 18.2 keV at the end of the SWS which means most of the particles have lost energy that amplified the RF signal. We set particles’ position monitor to observe the inset of electron cluster diagram from 3 to 50 ns which is about the same as is shown in Fig. 14a where the electron beam bunches very well rather than intercepting on the wall at the end of the SWS. So, we can conclude that our TWT can operate steadily during the 50 ns long simulation time.

By sweeping the frequency from 80 to 110 GHz with 18 integral frequencies, which is a long-time simulation (each frequency point should be calculated for one weeks), we obtain the output power–frequency and gain-frequency response of our high-power double-mode two-stage TWT as shown in Fig. 14b. We can see that the 3 dB bandwidth of about 29 GHz (81 GHz to 110 GHz) can be achieved. It is two modes (mode 1 from 81-103 GHz and mode 2 from 104-110 GHz) which could interact with the same energy electron particles obviously; compared to our previous work, the bandwidth in this paper is indeed expanded from 15 to 29 GHz, which is nearly twice than before^11^. It implies that when the TWT operates at the fundamental double modes rather than in the fundamental one mode, the beam-wave interaction bandwidth could be increased undoubtedly. The TWT we designed has a certain input signal. It could be possible to generate mode competitions and oscillations in TWT. But under the influence of the input signal and beam-wave interaction, the competitions at other frequencies and other modes are suppressed successfully, only signals of the same frequency as the input signal are amplified. As our hot simulation, there is no oscillation with the stable amplified output signals of each frequency from 80–110 GHz. The simulation results show that the output signal is stable and the frequency spectrum is pure, which means the oscillations are suppressed in our TWT. Thus, our double-mode two-stage a planar alignment plan alignment multiple-beam SDV-TWT can be used as the new scheme for the ultra-wide band and high output power in the millimeter and terahertz band in the future.

## Conclusion

This paper presents a thorough design, fabrication and analysis of a double-mode two-stage planar alignment three-pencil beam staggered double vane circuit in W-band. The simulation results show that it indeed has an ultra-wide beam-wave matching band and a very high interaction impedance. Two-stage SDV-SWS structure and an attenuator between two sections are adopted to suppress reflection and oscillation, and a simple input/output coupler without any taper is designed to get a high transmission performance. The cold test results of manufacturing SDV-SWS show that this TWT indeed can promote bandwidth as much as simulation results. 3D PIC simulation results predict that at 94 GHz our TWT can achieve a peak output power of 2120 W, a peak gain of 42.5 dB and an electron efficiency of 10.7% with a very short length of about 70 mm, and simultaneously can get a 3 dB bandwidth of 29 GHz. Anyway, the important thing is, the adoption of double-mode with matching of the same electron beam can indeed increase bandwidth, and the multiple pencil beam SDV-SWS can enhance the output power, gain, and growth rate in some extends. Also, the investigation of electron gun and optics system for such planar alignment three pencil beam have been performed by our group recently^29^. Such good ideas as double-mode and planar alignment multiple pencil beam have been used at the high terahertz band, e.g. 220 GHz and 340 GHz, some initial good results with simulation have been obtained for such schemes, and the ultra-wideband and high output can be achieved with SDV-TWT^13,14^. Therefore, it really has great potentials and values in our follow-up work at terahertz band in future.
